# Surgical management for follicular variant of papillary thyroid carcinoma

**DOI:** 10.18632/oncotarget.18525

**Published:** 2017-06-16

**Authors:** Jianing Tang, Deguang Kong, Lupin Bu, Gaosong Wu

**Affiliations:** ^1^ Department of Breast and Thyroid Surgery, Zhongnan Hospital of Wuhan University, Wuhan, Hubei, P.R. China; ^2^ Department of General Surgery, Zhongnan Hospital of Wuhan University, Wuhan, Hubei, P.R. China

**Keywords:** follicular variant of papillary thyroid carcinoma, surgery, SEER

## Abstract

**Background and Aims:**

For most patients with follicular variant of papillary thyroid carcinoma (FVPTC), surgery is required, while the surgical management remains controversial. We aim to further understanding of treatment of FVPTC and to determine whether specific features could be identified for the decision of surgical strategy.

**Materials and Methods:**

Data were obtained from the Surveillance, Epidemiology, and End Results (SEER) Program database during 2003 and 2013. 26700 patients were eligible and stratified by tumor size or extension. Survival rates were compared using multivariate Cox proportional hazard regressions.

**Results:**

Of the total death of 1041, 136 patients died from thyroid cancer. Most patients (79.1%) underwent total thyroidectomy while only a little part of patients (8.2%) underwent lobectomy. Patients receiving radioisotopes had significantly better overall survival (OS) (HR = 0.659, *P* < 0.001), but showed no differences on disease-specific survival (DSS). No statistical difference was found between total thyroidectomy and lobectomy in multivariate analysis when controlling for tumor size. While for tumor > 2 cm with extrathyroidal extension, lobectomy had significantly worse OS (aHR = 3.364, *P* = 0.010) and DSS (aHR = 5.494, *P* = 0.032) compared to total thyroidectomy. Multivariate analysis demonstrated that advanced age, male, higher grade, extrathyroidal extension, lymph nodes metastases and distant metastases had negative effects on OS and DSS controlling for the remaining variables (each *P* < 0.05).

**Conclusions:**

The results of our study revealed total thyroidectomy could benefit the survival for patients whose tumors > 2 cm with extrathyroidal extension, total thyroidectomy should be recommended for those patients. Lots of factors should be taken into consideration on the decision of surgical treatment.

## INTRODUCTION

The incidence of follicular variant of papillary thyroid cancer (FVPTC) has increased rapidly during the past decade, accounting for 24%–33% of papillary thyroid cancer (PTC), a more recent study indicated that the figure had risen to 41% [1, 2]. Patients of thyroid cancer doubled from 1997 to 2007, climbing to 62450 in 2015 in American, well differentiated thyroid tumors take up approximately 90% of thyroid malignance, especially PTC (> 70%) [3–6].

Despite of the rapid increase of FVPTC, the clinical behavior and risk factors are under debate. FVPTC is defined as one of indolent tumors having excellent prognosis. Cytologically, FVPTC almost completely consists of follicular architectural pattern with nuclear features of PTC. It was first described by Crile and Hazard [7, 8]. According to histological growth patterns, FVPTC is classified into two major classes: nonencapsulated FVPTC which often infiltrate the surrounding tissues, has extrathyroidal extension and more frequent lymph node metastases, thus the diagnosis is usually clear, while the encapsulated FVPTC behave more indolently, similar to follicular adenomas, we can only diagnose by the nuclear features [8–10]. The clinical behavior of FVPTC is considered as more aggressive than pure PTC, and it is suggested having a tendency for pulmonary metastases [11, 12], but no previous studies proved that the pulmonary metastases appeared more common than PTC. Recent study shows that the rate of extrathyroidal extension and lymph node metastases is much lower compared with PTC, but higher than follicular thyroid carcinoma (FTC). The lymph node metastases do not impact survival in patients having FVPTC but influence the recurrence [13], while it is inverse in other studies [3]. Distant metastases appear uncommon in FVPTC, the rate in PTC is half but doubled in FTC. It is reported that the tumor size of FVPTC is smaller and it is often encapsulated. There are more capsular invasions in the FVPTC than that in the PTC. The mortality is similar between FVPTC and PTC while much higher in FTC [3].

Even now lots of researches have been undertaken on the clinical behavior of FVPTC, the option of surgical management remains controversial. Total thyroidectomy and lobectomy are two primary choices for patients with FVPTC. Some physicians suggest a total thyroidectomy for these tumors size ≥ 1.5 cm [14]. There are also recommendations that as for the encapsulated, noninvasive FVPTC, a lobectomy is enough [2, 15]. The findings of Rosai et al. suggested that patients with noninvasive FVPTC who underwent lobectomy could decrease the recurrence [8]. Some other experts believed the patients with FVPTC and PTC should receive the same treatment strategy [5, 16]. The operative approach of FVPTC is not consistent yet. In the current study, according to a small size sample, the overall survival displays no difference between patients who underwent total or subtotal thyroidectomy [5]. To evaluate the impact of operative treatment and mortality of FVPTC, a large population of patients with a long-term follow-up is needed, this could be the raison for the lack of consensus on surgical treatment. The American Thyroid Association (ATA) Management Guidelines recommends a total thyroidectomy if tumor treasures > 4 cm, for low-risk carcinomas > 1 cm and < 4 cm, the treatment preferred is thyroid lobectomy, and tumors < 1 cm, lobectomy alone is sufficient, but this recommendation is from moderate-quality evidence [17]. No study of large population-based cohorts analysis has been reported that a more aggressive surgical approach could improve the OS and DSS on patients with FVPTC. Our study aims to determine whether a more aggressive surgical approach is required for FVPTC patients with specific features and to investigate the outcomes of surgical managements on FVPTC using the Surveillance, Epidemiology, and End Results (SEER) Program database of the National Cancer Institute.

## RESULTS

### Patient characteristics

In the SEER database, a total of 29519 patients were diagnosed of FVPTC from 2003 to 2013. After the exclusion of the patients who were not eligible for our study (patients without tumor information, or survival times were unknown), 26700 patients were selected for the further analyses. The patients were classified into four subgroups based on tumor size as follows: 10160 patients whose tumor size was smaller than 1 cm (38.1%), 7749 between 1cm and 2 cm (29%), 6254 between 2.1 cm and 4 cm (23.4%), 2537 lager than 4 cm (9.5%).

Of the total death of 1041, 136 patients died from thyroid cancer. In the 136 thyroid cancer specific mortality, 77 patients had extrathyroidal extension (56.6%), lymph nodes metastases appeared in 44 patients (32.4%), 41 patients were diagnosed with distant metastases (30.1%).

We summarized different characteristics such as demographics, treatments, clinicopathologic features and outcomes in Table 1. FVPTC seemed to occur more often in patients > 20 years (98.6%), furthermore, tumors in male had a tendency for larger than 4cm (15.9%) compared to female (7.8%). In general, a larger tumor had a more advanced grade, extrathyroidal extension of FVPTC was more frequently observed in size > 4 cm, lymph nodes metastases and distant metastases were rarely appeared in FVPTC patients. As for treatment strategy, patients of all tumor sizes were strongly advised of a total thyroidectomy (78.9%), other surgical treatments were used more often for tumors < 1 cm. Tumors < 1 cm received less radio-therapy, while tumors larger than 1 cm were more likely to be given radioisotopes.

**Table 1:** Patient characteristics within subgroups

Variables	≤ 10 mm *N* = 10160 (%)	11–20 mm *N* = 7749 (%)	21–40 mm *N* = 6254 (%)	> 40 mm *N* = 2537 (%)	*P* value*
**Median follow-up(months)**	41.94	43.97	45.26	42.66	
**Age at diagnosis, y**					*P* < 0.001
**< 20**	68 (0.7)	111 (1.4)	138 (2.2)	59 (2.3)	
**20–44**	2859 (31.8)	2662 (34.4)	2502 (40.0)	959 (37.8)	
**45–64**	5203 (51.2)	3653 (47.1)	2627 (42.0)	981 (37.8)	
**≥ 65**	2030 (20.0)	1323 (17.1)	987 (15.8)	538 (21.2)	
**Sex**					*P* < 0.001
**Male**	1764 (17.4)	1513 (19.5)	1507 (24.1)	902 (35.6)	
**Female**	8396 (82.6)	6236 (80.5)	4747 (75.9)	1635 (64.4)	
**Race**					*P* < 0.001
**white**	8546 (84.1)	6433 (83.0)	5053 (80.8)	1904 (75.0)	
**Black**	794 (7.8)	545 (7.0)	526 (8.4)	362 (14.3)	
**Chinese**	86 (0.8)	95 (1.2)	80 (1.3)	28 (1.1)	
**Japanese**	34 (0.3)	35 (0.5)	27 (0.4)	7 (0.3)	
**Filipino**	179 (1.8)	167 (2.2)	174 (2.8)	88 (3.5)	
**Other**	457 (4.5)	434 (5.6)	349 (5.6)	134 (5.3)	
**Unknown**	64 (0.6)	40 (0.5)	45 (0.7)	14 (0.6)	
**Region**					*P* < 0.001
**East**	4272 (42.0)	3242 (41.8)	2475 (39.6)	973 (38.4)	
**Northern Plains**	971 (9.6)	714 (9.2)	640 (10.2)	315 (12.4)	
**Pacific Coast**	4172 (41.1)	3265 (42.1)	2739 (43.8)	1120 (44.1)	
**Southwest**	739 (7.3)	523 (6.7)	391 (6.3)	128 (5.0)	
**Alaska**	6 (0.1)	5 (0.1)	9 (0.1)	1 (0.0)	
**Laterality**					0.939
**Right**	203 (2.0)	146 (1.9)	125 (2.0)	50 (2.0)	
**Left**	146 (1.4)	112 (1.4)	80 (1.3)	34 (1.3)	
**Bilateral**	38 (0.4)	43 (0.6)	29 (0.5)	12 (0.5)	
**Pared site**	2 (0.0)	3 (0.0)	1 (0.0)	1 (0.0)	
**unknown**	9771 (96.2)	7445 (96.1)	6019 (96.2)	2440 (96.2)	
**Grade**					*P* < 0.001
**Well**	1600 (15.7)	1256 (16.2)	1021 (16.3)	407 (16.0)	
**Moderately**	270 (2.7)	312 (4.0)	279 (4.5)	118 (4.7)	
**Poorly**	17 (0.2)	41 (0.5)	60 (1.0)	76 (3.0)	
**Undifferentiated**	4 (0.0)	4 (0.1)	15 (0.2)	13 (0.5)	
**Unknown**	8269 (81.4)	6136 (79.2)	4879 (78.0)	1923 (75.8)	
**Surgery**					*P* < 0.001
**Total thyroidectomy**	7283 (71.7)	6528 (84.2)	5166 (82.6)	2084 (82.1)	
**Less than a lobe**	109 (1.1)	42 (0.5)	29 (0.5)	17 (0.7)	
**Lobectomy and/or isthmectomy**	202 (2.0)	79 (1.0)	64 (1.0)	27 (1.1)	
**Lobectomy ONLY**	1204 (11.9)	426 (5.5)	400 (6.4)	160 (6.3)	
**Isthmectomy ONLY**	15 (0.1)	13 (0.2)	4 (0.1)	1 (0.0)	
**Lobectomy WITH isthmus**	734 (7.2)	269 (3.5)	234 (3.7)	99 (3.9)	
**Removal of a lobe and partial removal of the contralateral lobe**	101 (1.0)	49 (0.6)	50 (0.8)	13 (0.5)	
**Subtotal or near total thyroidectomy**	429 (4.2)	263 (3.4)	235 (3.8)	98 (3.9)	
**None**	26 (0.3)	32 (0.4)	24 (0.4)	28 (1.1)	
**Unknown**	57 (0.6)	48 (0.6)	48 (0.8)	10 (0.4)	
**Radiotherapy**					*P* < 0.001
**None**	6996 (68.9)	3123 (40.3)	2033 (32.5)	814 (32.1)	
**Radioisotopes**	2859 (28.1)	4307 (55.6)	3924 (62.7)	1582 (62.4)	
**Beam radiation**	43 (0.4)	72 (0.9)	66 (1.1)	42 (1.7)	
**Combination of beam with implants or isotopes**	5 (0.0)	14 (0.2)	19 (0.3)	10 (0.4)	
**Radiation, NOS method or source not specified**	10 (0.1)	19 (0.2)	15 (0.2)	4 (0.2)	
**Radioactive implants**	44 (0.4)	71 (0.9)	65 (1.0)	27 (1.1)	
**Unknown**	203 (2.0)	143 (1.8)	132 (2.1)	58 (2.3)	
**Extension**					*P* < 0.001
**Single invasive tumor confined to thyroid**	5577 (54.9)	2866 (37.0)	2299 (36.8)	757 (29.8)	
**Multiple foci confined to thyroid**	3252 (32.0)	2696 (34.8)	1945 (31.1)	634 (25.0)	
**Into thyroid capsule**	405 (4.0)	625 (8.1)	755 (12.1)	413 (16.3)	
**Beyond thyroid capsule**	515 (5.1)	1124 (14.5)	849 (13.6)	524 (20.7)	
**Unknown**	411 (4.0)	438 (5.7)	406 (6.5)	209 (8.2)	
**Lymph nodes metastases**					*P* < 0.001
**None**	9267 (91.2)	6462 (83.4)	5188 (83.0)	2085 (82.2)	
**Yes**	654 (6.4)	997 (12.9)	786 (12.6)	332 (13.1)	
**Unknown**	239 (2.4)	290 (3.7)	280 (4.5)	120 (4.7)	
**Distant metastases**					*P* < 0.001
**None**	10043 (98.8)	7608 (98.2)	6080 (97.2)	2406 (94.8)	
**Yes**	27 (0.3)	51 (0.7)	82 (1.3)	90 (3.5)	
**Unknown**	90 (0.9)	90 (1.2)	92 (1.5)	41 (1.6)	
**Status**					*P* < 0.001
**Alive**	9787 (96.3)	7479 (96.5)	6014 (96.2)	2379 (93.8)	
**Dead**	373 (3.7)	270 (3.5)	240 (3.8)	158 (6.2)	
**Thyroid cancer**	18 (0.2)	26 (0.3)	41 (0.7)	51 (2.0)	
**other**	355 (3.5)	244 (3.1)	199 (3.2)	107 (4.2)	

*P values calculated by Pearson Chi squared testing.

y: years.

### Survival analysis

**Figure 1:**
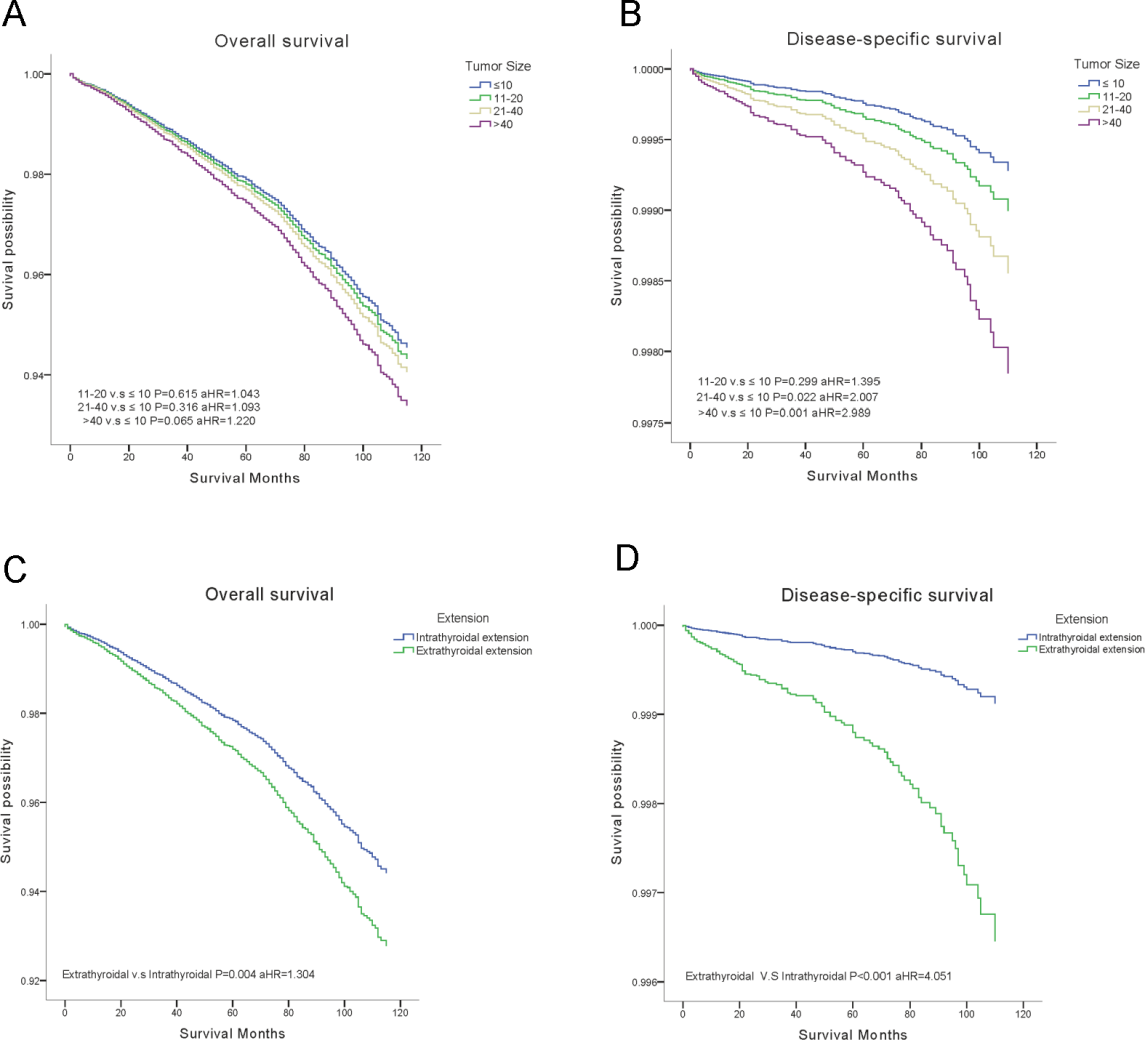
Overall survival (OS) and disease-specific survival (DSS) curves of multivariate Cox analysis (**A**) OS is based on tumor size. (**B**) DSS is based on tumor size. (**C**) OS is based on tumor extension. (**D**) DSS is based on tumor extension.

Outcomes of OS and DSS were obtained using the multivariate Cox analysis. Cox proportion hazards models were applied to understand the clinical significance of the variables in Table 2. The final model showed the strength of relative risk. Interestingly, increased tumor size only influenced DSS, tumors > 4 cm demonstrated the greatest decrease in DSS (aHR = 2.989, *P* = 0.001) (Figure 1A, 1B). Extraexthyroidal extension had negative effects both in OS (aHR = 1.304, *P* = 0.004) and DSS (aHR = 4.051, *P* < 0.001) (Figure 1C, 1D).

**Table 2:** Cox proportional hazards regression model analysis of overall survival (OS) and disease-specific survival (DSS)

Variables	OS		DSS	
	aHR (95% CI)	*P*-value	aHR (95% CI)	*P*-value
**Age at diagnosis, y**				
**< 20**	Reference		Reference	
**20–44**	1.005 (0.316,3.199)	0.993	0.083 (0.005,1.345)	0.080
**45–64**	4.280 (1.372,13.357)	0.012	3.907 (0.532,28.692)	0.180
**≥ 65**	15.848 (5.086,49.388)	*P* < 0.001	10.063 (1.372,73.807)	0.023
**Sex**				
**Male**	Reference		Reference	
**Female**	0.568 (0.499,0.647)	*P* < 0.001	0.548 (0.382,0.786)	0.001
**Race**				
**White**	Reference		Reference	
**Black**	1.571 (1.286,1.919)	*P* < 0.001	1.814 (1.003,3.281)	0.049
**Grade**				
**Well**	Reference		Reference	
**Moderately**	1.222 (0.845,1.768)	0.287	0.596 (0.162,2.184)	0.434
**Poorly**	2.007 (1.269,3.173)	0.003	4.936 (2.106,11.566)	*P* < 0.001
**Undifferentiated**	10.128 (6.086,16.853)	*P* < 0.001	12.963 (5.358,31.361)	*P* < 0.001
**Surgery**				
**Total thyroidectomy**	Reference		Reference	
**Less than a lobe**	1.544 (0.921,2.588)	0.100	4.719 (1.671,13.330)	0.003
**Lobectomy ONLY**	1.039 (0.827,1.306)	0.742	1.325 (0.591,2.971)	0.495
**Lobectomy WITH isthmus**	0.870 (0.650,1.166)	0.352	0.234 (0.032,1.702)	0.151
**Removal of a lobe and partial removal of the contralateral lobe**	0.803 (0.400,1.615)	0.539	0.947 (0.131,6.858)	0.957
**Subtotal or near total thyroidectomy**	0.980 (0.734,1.308)	0.892	0.539 (0.170,1.710)	0.294
**None**	5.840 (4.056,8.409)	*P* < 0.001	2.491 (1.120,5.542)	0.025
**Radiotherapy**				
**None**	Reference		Reference	
**Radioisotopes**	0.659 (0.573,0.758)	*P* < 0.001	0.794 (0.529,1.193)	0.267
**Tumor size(mm)**				
**≤ 10**	Reference		Reference	
**11–20**	1.043 (0.885,1.229)	0.615	1.395 (0.744,2.615)	0.299
**21–40**	1.093 (0.918,1.302)	0.316	2.007 (1.107,3.615)	0.022
**> 40**	1.220 (0.988,1.505)	0.065	2.989 (1.290,5.618)	0.001
**Extension**				
**Single invasive tumor confined to thyroid**	Reference		Reference	
**Multiple foci confined to thyroid**	0.997 (0.836,1.142)	0.768	0.679 (0.349,1.320)	0.254
**Into thyroid capsule**	1.040 (0.807,1.580)	0.764	1.554 (0.724,3.336)	0.258
**Beyond thyroid capsule**	1.297 (1.065,1.580)	0.010	3.753 (2.154,6.538)	*P* < 0.001
**Lymph nodes metastases**				
**None**	Reference		Reference	
**Yes**	1.357 (1.112,1.657)	0.003	1.983 (1.278,3.077)	0.002
**Distant metastases**				
**None**	Reference		Reference	
**Yes**	3.956 (2.993,5.230)	*P* < 0.001	9.903 (6.252,15.687)	*P* < 0.001

*P values calculated by multivariate Cox analysis.

aHR: adjusted hazard ratio (adjusted for age at diagnosis, sex, race, grade, region, tumor size, laterality, extension, lymph nodes metastases, distant metastases, radiotherapy, and surgery).

CL: confidence interval.

**Figure 2:**
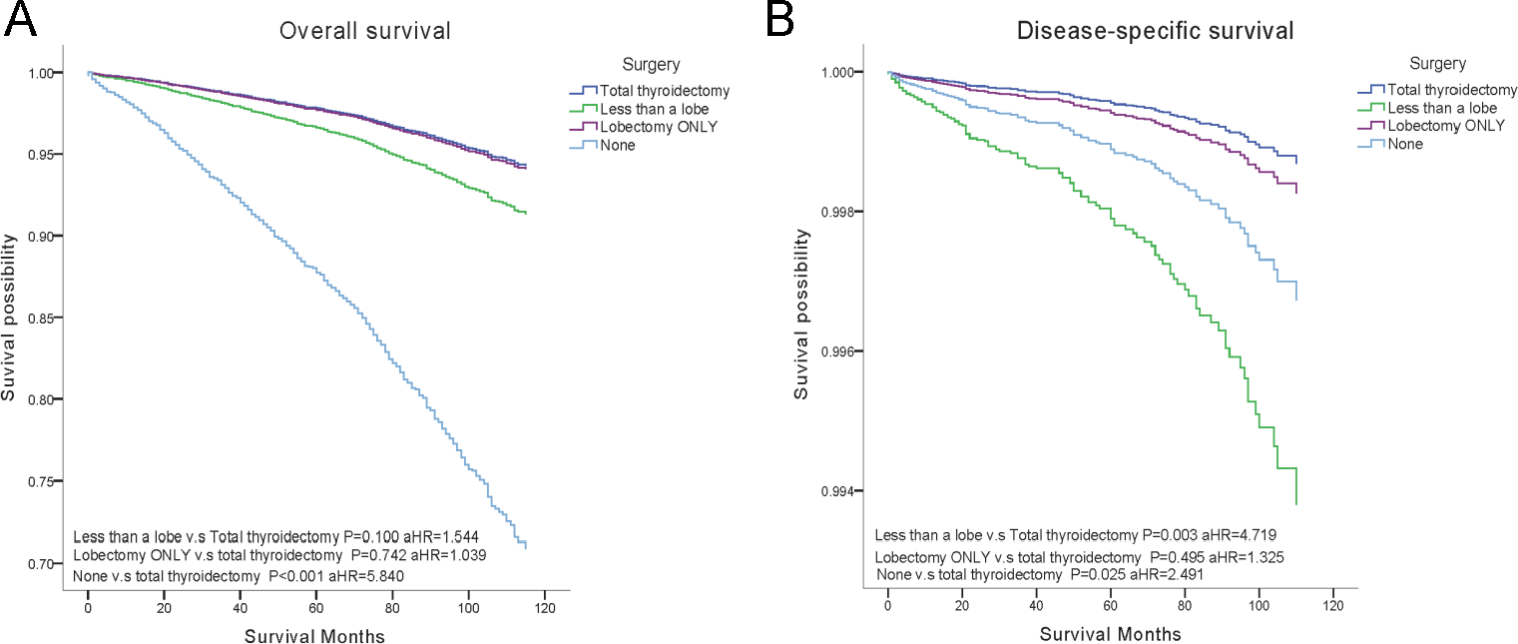
Overall survival (OS) and disease-specific survival (DSS) curves of multivariate Cox analysis (**A**) OS is based on surgical treatment. (**B**). DSS is based on surgical treatment.

Patients who underwent radioisotopes had significantly better OS (aHR = 0.659, *P* < 0.001), but showed no differences on DSS, even by subgroup analysis, DSS was not improved by radioisotopes. Surgical treatments did not reveal statistical differences in OS (ten-year survival rates of total thyroidectomy 96.4%, lobectomy 95.9%), except that removal of less than a lobe displayed significantly decreased DSS (aHR = 4.719, *P* = 0.003) (Figure 2).

### Subgroup analysis of surgical effects

**Figure 3:**
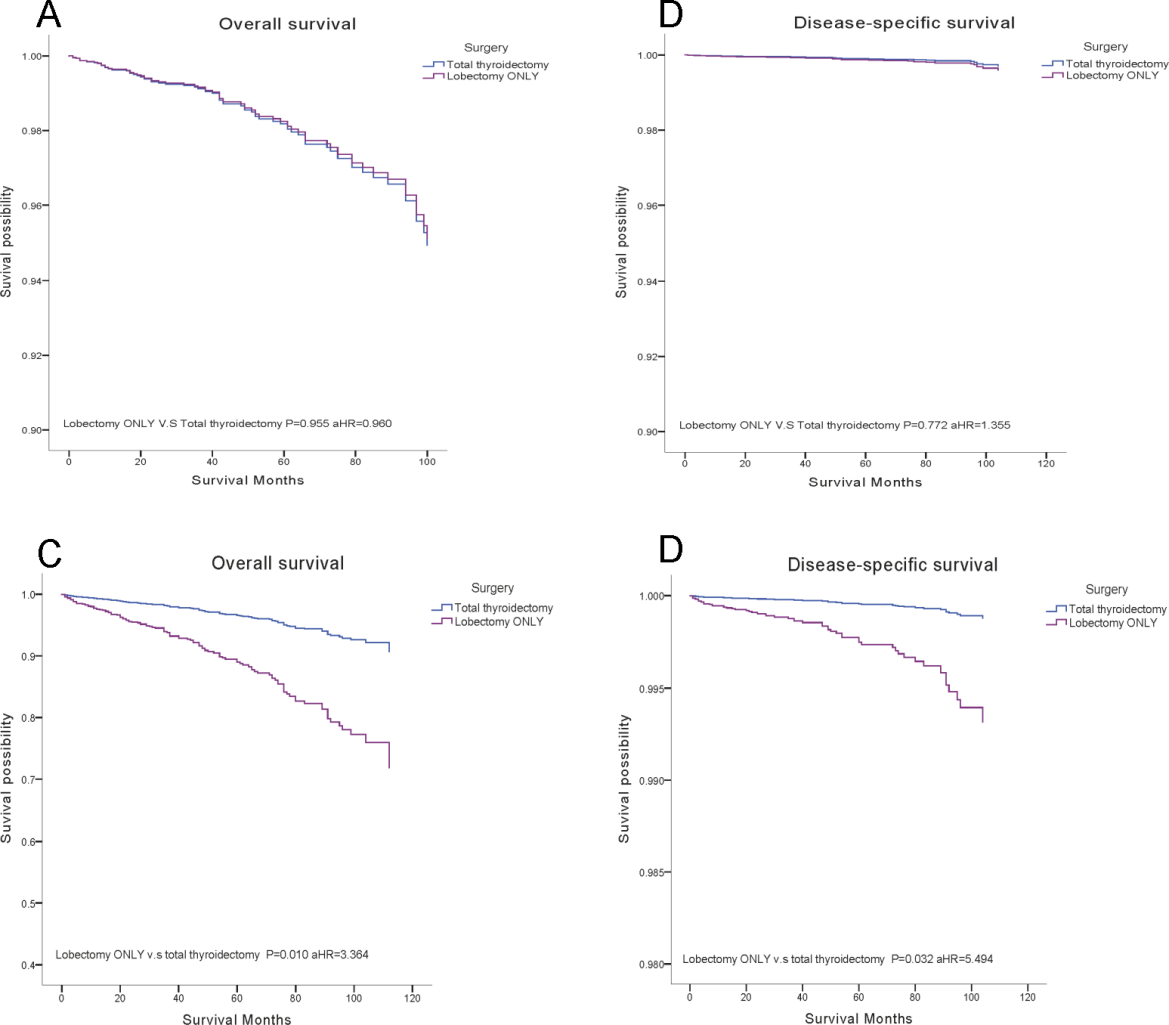
Overall survival (OS) and disease-specific survival (DSS) curves of multivariate Cox analysis in subgroup analysis (**A**) OS is based on surgical treatment in tumor < 2 cm with extrathyroidal extension. (**B**) DSS is based on surgical treatment in tumor < 2 cm with extrathyroidal extension. (**C**) OS is based on surgical treatment in tumor >2 cm with extrathyroidal extension. (**D**) DSS is based on surgical treatment in tumor > 2 cm with extrathyroidal extension.

We stratified the cases into four subgroups on the basis of tumor size (≤ 1 cm, 1.1 to 2.0 cm, 2.1 to 4.0 cm, > 4.0 cm). A multivariate analysis was performed to investigate the effects of surgical treatments (our attention focused on the lobectomy and total thyroidectomy) (Supplementary Figures 1, 2). During period of the follow-up, results indicated that a more aggressive surgical approach will not affect the survival, there were no statistical differences between total thyroidectomy and lobectomy, noteworthy is the surgical therapies for tumor > 2 cm with extrathyroidial extension, surgical therapy of lobectomy had significantly decreased OS (aHR = 3.364, *P* = 0.010) and DSS (aHR = 5.494, *P* = 0.032) compared to total thyroidectomy, but the same result was not found in tumor < 2 cm with extrathyroidial extension (Figure 3).

## DISCUSSION

FVPTC is the major subtype of PTC and is considered as an indolent cancer. In our retrospective analysis based on a large population, we studied the potential risk factors for long-term survival. In previous studies, age, tumor size, lymph nodes metastases, extrathyroidal extension and distant metastases were acknowledged strong predictors of survival rate [3]. In our study, we found that tumor size only decreased DSS, this may due to the death caused by the thyroid was in a minority (136 compared to 1041), OS was not influenced significantly by tumor size. Interestingly, female had a better OS and DSS, the essentially possible explanation of the disparities observed between men and women including differential screening, gender specific behavioral differences and biological sex differences. It is of note that in female group, tumors > 4 cm accounted for 7.8% but the rate rose to 15.8% in male, this may be owing to self-protection awareness of women, they would be diagnosed earlier, thus female patients were more likely to be diagnosed at early stage. Men were more likely died from other reasons. In addition, women were regarded as having greater longevity compared with men regardless of cancer status. Therefore, OS and DSS were decreased in male. To our surprise is that radioisotopes only benefited OS. The reason could be related to the selection bias: the patients receiving radioisotopes had larger tumor size, they were more often in the advanced stage. Radioisotopes could not improve the already poor DSS. While they were more likely to have better surveillance, keep in touch with their physicians, thereby improved their OS.

The results enforce us to reconsider the surgical recommendations, the 2015 ATA guidelines advocated a near-total or total thyroidectomy for tumor > 4 cm, while for low risk papillary and follicular carcinomas > 1 cm and < 4 cm without extrathyroidal extension, and without clinical evidence of any lymph node metastases, thyroid lobectomy alone is enough, and we should choose thyroid lobectomy as the initial surgical procedure for tumor intrathyroidal < 1 cm (Table 3) [17]. Our study found that surgical treatment based on tumor size did not influence the survival of FVPTC, similar results have been found in previous reports on PTC, in these studies, no significant benefit on survival was demonstrated between thyroidectomy and lobectomy [18, 19]. In contrast, other findings revealed that lobectomy could cause an increased rate of recurrence and death than total thyroidectomy on PTC [20]. Despite the studies on PTC, there are no large population-based researches with respect to FVPTC Supplement Table 1. In current studies, it is agreed that FVPTC and PTC should be treated with the similar surgical strategy [5, 16], but a recent study reveal that FVPTC patients who had extension of extrathyroid, the disease-specific mortality is higher than that of PTC, and total thyroidectomy did not improve the survival of FVPTC [3]. As FVPTC is divided into encapsulated and non-encapsulated, many experts today suggested a lobectomy for patients with noninvasive, encapsulated FVPTC. And for these low risk patients with excellent prognosis, the extent of surgery did not impact the survival [21, 22]. It is reported that for these low risk tumors < 1 cm, as is showed in our study, lobectomy alone is sufficient, a more aggressive approach demonstrates no statistical differences, while for high risk factors as tumors > 4 cm, a total thyroidectomy is needed [23]. Recent studies only stratified patients based on tumor size, while whether the tumor invades extra capsule is another consideration influencing our option of treatment given for patients with FVPTC. To find an answer to this debate, we performed a further analysis based on the extension of tumor (intrathyroidal and extrathyroidal), and the cut-off for tumor size was 2 cm. For tumors < 2 cm, OS and DSS of total thyroidectomy did not differ from that of lobectomy both in intrathyroidal and extrathyroidal subgroups, thus lobectomy for these patients with tumor < 2 cm may be sufficient. However, for tumors > 2 cm, lobectomy had a significantly decrease on OS and DSS in subgroup of extrathyroidal, thereby total thyroidectomy should be recommended. This result was not shown in tumor > 2 cm with intrathyroidal extension subgroup, total thyroidectomy should not be the initial treatment. The results of our study make us to reconsider the surgical treatment for FVPTC patients, tumor size and extension are two main factors affect our option for surgery. As is reported in previous studies, size and extension were two risk features predicting the surgical outcomes [23]. In our study, extrathyroidial extension is a strong predictor reminding a total thyroidectomy for tumor larger than 2 cm. While for tumor < 2 cm, whether or not had extrathyroidial extension dose not influence our surgical options, a more aggressive surgical approach does not benefit the OS and DSS, lobectomy should be the initial treatment. And lobectomy has potential benefits like reducing the injury of laryngeal nerve and other complications as well as a higher potential of recurrence, therefore, we must choose the most suitable surgical treatment for patients [24]. Our study revealed that total thyroidectomy benefits the survival for tumors > 2 cm with extrathyroidal extension based on a large population analysis using the SEER database.

**Table 3:** ATA guidelines for surgical treatment

Tumor characteristics	Surgical treatment
Thyroid cancer > 4 cm	Near-total or total thyroidectomy
Gross extrathyroidal extension	Near-total or total thyroidectomy
Clinically apparent metastatic disease to nodes or distant sites	Near-total or total thyroidectomy
Thyroid cancer > 1 cm and < 4 cm without extrathyroidal extension, and without clinical evidence of any lymph node metastases (cN0)	Near-total or total thyroidectomy or lobectomy
Low risk papillary and follicular carcinomas	Lobectomy
Thyroid cancer < 1 cm without extrathyroidal extension and cN0	Lobectomy

Our study has several limitations, because of the indolent nature of FVPTC, it is difficult to study with large, prospective trials, and the database is potentially biased by prejudice and treatment practices. In addition, the SEER database missed lots of information, we could not put TNM stage into analysis because of this problem. Molecular markers were not included in SEER, it is agreed molecular markers would also affect the option of surgical approach [13]. SEER database did not capture the information on recurrence records, due to the rare specific mortality of FVPTC, recurrence is more meaningful than death. This is the reason why previous studies failed to demonstrate a more aggressive surgical approach is better for survival. Another limitation of our analysis is that we only put extension and tumor size into consideration. In fact, age, grade, metastases, even DNA ploidy are factors we should take into account, these are prognostic features to identify patients’ survival [23]. Despite many institutions have published the risk stratification systems for predicting surgical outcomes, challenges still exist in stratifying patients into low risk and high risk groups [17, 25–27]. Tumor size is considered as the main factor for classifying prognostic group, the thresholds of size remain controversial from 1 cm to 5 cm, and only small patient cohorts were included in these systems [28, 29].

With the excellent prognosis of FVPTC, lobectomy should be the initial surgical procedure for tumors < 2 cm or > 2 cm without extrathyroidal extension, this kind of FVPTC is regarded as low risk. Tumors > 2 cm with extrathyroidal extension should undergo total thyroidectomy which could benefit the survival, tumors > 4 cm is not the strong predictor for total thyroidectomy. While in our study, most patient underwent total thyroidectomy (78.9%), even for patients with tumor < 1 cm (71.7%). Although for these low risk patients, lobectomy alone is enough, surgeons preferred to offer total thyroidectomy, this may partially because of the rates of revision surgery and the risk associated with re-operation. Thus a large population of FVPTC patients were over treatment. Another reason for recommending total thyroidectomy is that radioactive iodine can only be administered to patients after total thyroidectomy. While thyroid lobectomy can reduce the laryngeal nerve injury and other complications, there is also higher chance of further surgery in the future. In conclusion, each patient should be treated individually, cancer characteristics including size, extension, metastases, and patient factors such as age, race, even profession are elements influencing our choice of surgical strategy for patients.

### MATERIALS AND METHODS

This is a population-based cohort analysis using data from the SEER program database between 2003 and 2013 provided by the National Cancer Institute. Patients diagnosed with FVPTC were identified using histopathology codes of the International Classification of Diseases for Oncology, 3rd edition (ICD-O-3): 8340/3 (Papillary carcinoma, follicular variant).

We excluded cases that survival months were unknown or tumor size information was blank. In order to emphasize our study on the effects of surgical treatments for different tumor sizes in patient with FVPTC, tumor size cutoffs were stratified according to previous studies and tumor-node-metastasis (TNM) staging systems as follows: tumors ≤ 1.0 cm, 1.1 to 2.0 cm, 2.1 to 4.0 cm and lager than 4 cm.

Our analysis included demographic variables: sex (male and female), age at diagnosis (< 20, 20–44, 45–64, ≥ 65 years), race (white, black, Chinese, Japanese, Filipino, other and unknown), region (East, Northern Plains, Pacific Coast, Southwest and Alaska). Cancer characteristics were separated by grade (well, moderately, poorly, undifferentiated, unknown), laterality (right, left, Paired site, unknown), tumor extension (code 100: single invasive tumor confined to thyroid; code 200: multiple foci confined to thyroid; code 400: into thyroid capsule, but not beyond; codes 450, 480, 500, 520, 550, 600, 620, 650, 700, 730, 800: extension beyond thyroid capsule; and unknown. Codes 100, 200, 400 were invasive intrathyroidal extension, other codes regarded as extrathyroidal extension), lymph nodes metastases (code 000: no lymph nodes metastases, codes 120, 135, 155, 158, 160: FVPTC with lymph nodes metastases), distant metastasis (code 00: no distant metastasis; code 12, 40, 51: distant metastases, unknown). Treatment characteristics included surgery therapy (code 00: no surgery of primary site, code 25 to 27: removal of less than a lobe, code 20: lobectomy and/or isthmectomy, code 21: Lobectomy only, code 22: isthmectomy only, code 23: lobectomy with isthmus, code 30: removal of a lobe and partial removal of the contralateral lobe, code 40: subtotal or near total thyroidectomy, code 50: total thyroidectomy and code 80 to 90: unknown), receipt of radiationtherapy (none, radioisotopes, beam radiation, combination of beam with implants or isotopes, radiation with not otherwise specified (NOS) method or source not specified, radioactive implants, unknown). All the variables were defined using the SEER specific codes.

The OS and DSS were two main outcomes in our study estimated using multivariate Cox proportional hazard regressions. Potential risk factors were selected based on ATA risk stratification system [17], the strength of relative risk was assessed. We tested the effects of tumor size and surgical treatments on OS and DSS controlling for the remaining variables, surgical treatments were then tested within each size-stratified subgroups (≤ 1 cm, 1.1 cm to 2 cm, 2.1 cm to 4 cm, > 4 cm). A Cox proportion hazards model was used to evaluate the relative risk of the factors on OS and DSS. Hazard ratios (HR) with 95% confidence intervals were obtained, any HR > 1.0 showed an increased risk of death. A *P* value < 0.05 was considered statistically significant and all tests were two-sided. All statistical analyses were performed using SPSS 19.0 (IBM Corporation, Armonk, NY).

## SUPPLEMENTARY MATERIALS FIGURES AND TABLE


