# Supplementary material for: Surgical management for follicular variant of papillary thyroid carcinoma
Source: Oncotarget. 2017 Jun 16;8(45):79507–16. doi: 10.18632/oncotarget.18525 (PMC5668063; doi:10.18632/oncotarget.18525)
Supplement: Supplementary file 1 [file oncotarget-08-79507-s001.pdf]

Surgical management for follicular variant of papillary thyroid carcinoma

Supplementary Materials

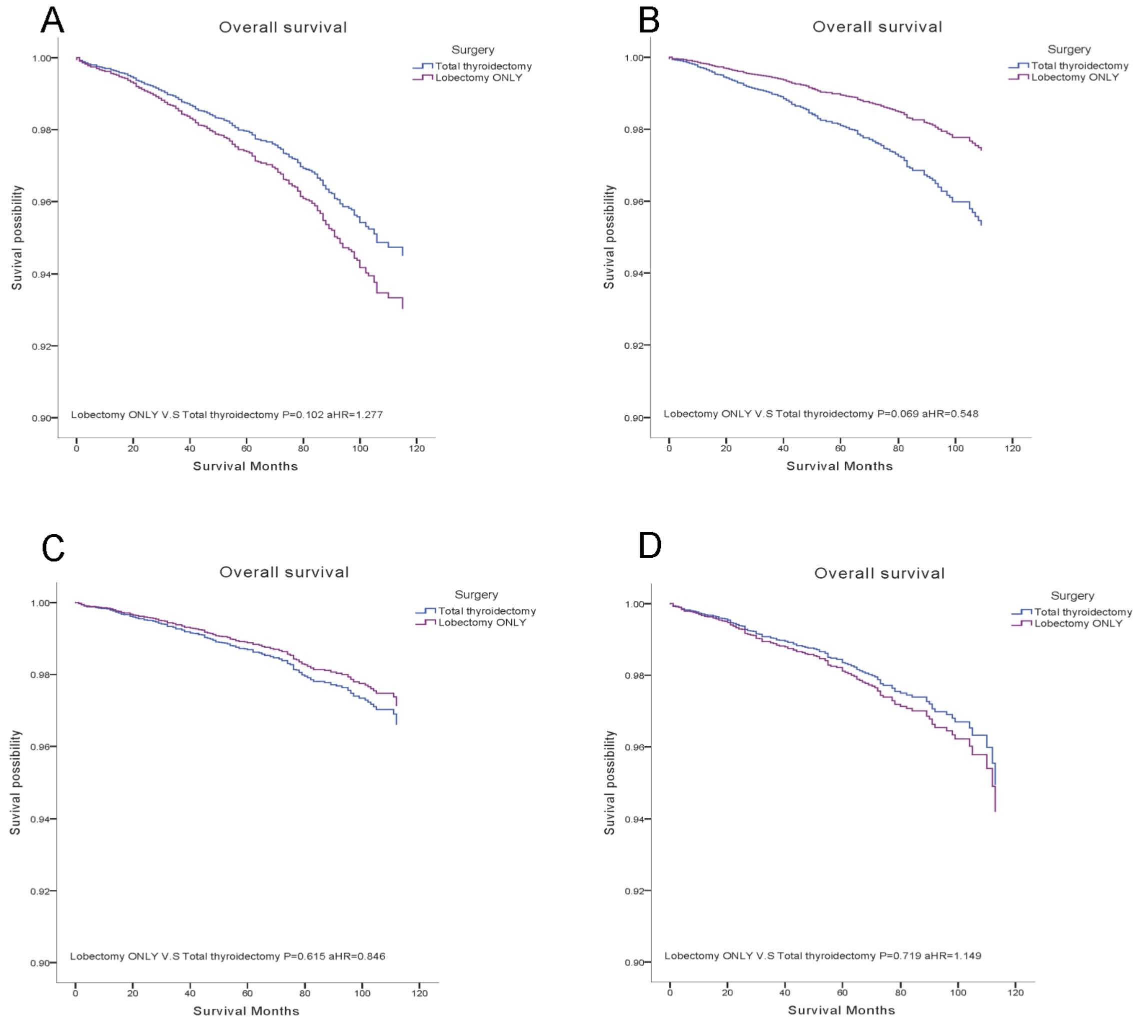

Supplementary Figure 1: Overall survival (OS) curves of multivariate Cox analysis in subgroup analysis. (**A**) OS is based on surgical treatment in tumor ≤ 1 cm. (**B**) OS is based on surgical treatment in tumor > 1 cm and ≤ 2 cm. (**C**) OS is based on surgical treatment in tumor > 2 cm and ≤ 4 cm. (**D**) OS is based on surgical treatment in tumor > 4 cm.

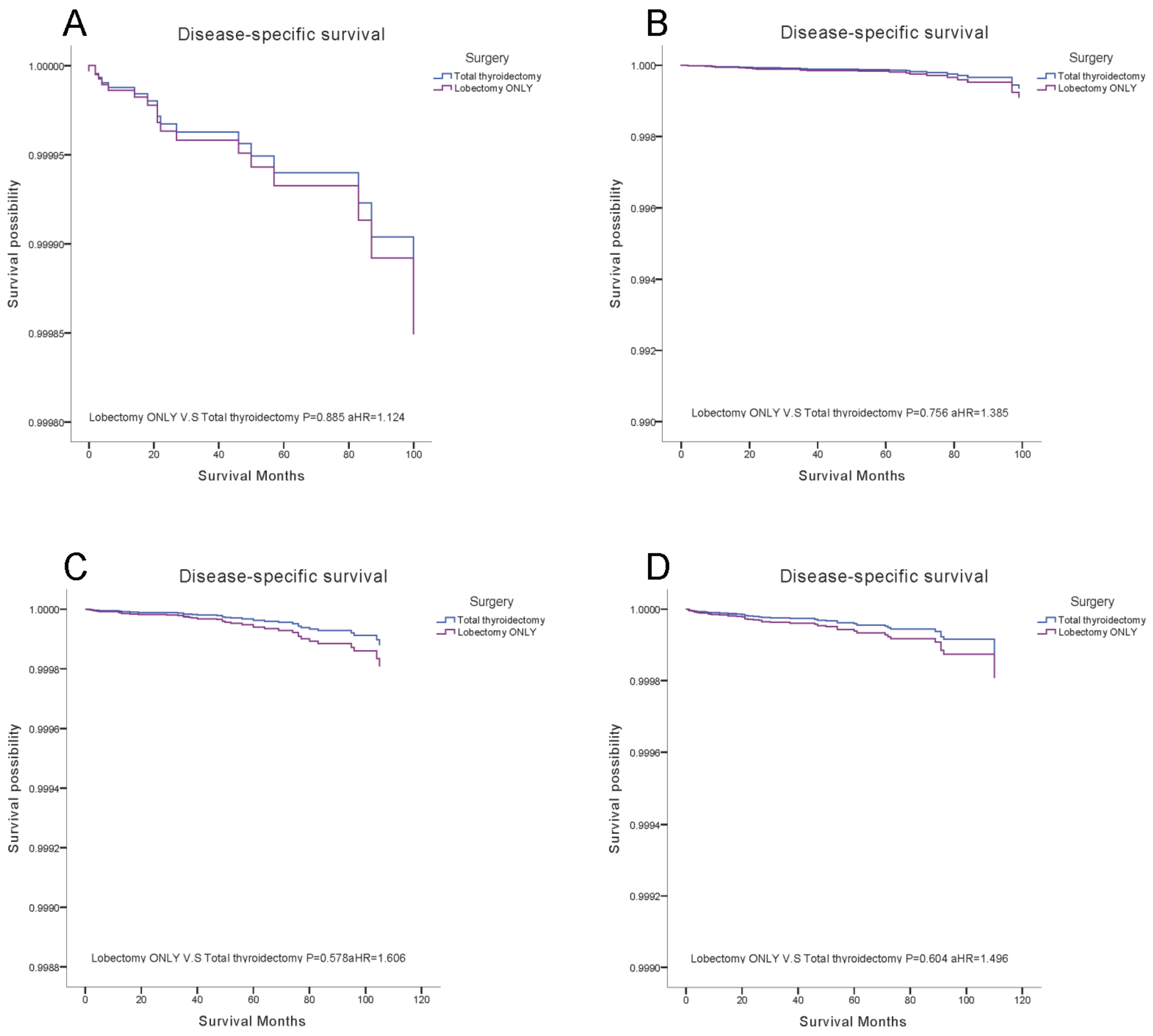

Supplementary Figure 2: Disease-specific survival (DSS) curves of multivariate Cox analysis. (**A**) DSS is based on surgical treatment in tumor ≤ 1 cm. (**B**) DSS is based on surgical treatment in tumor > 1 cm and ≤ 2 cm. (**C**) DSS is based on surgical treatment in tumor > 2 cm and ≤ 4 cm. (**D**) DSS is based on surgical treatment in tumor > 4 cm.

Supplementary Table 1: Summary of analysis of surgical treatment on thyroid cancer

Study	*N*	Histology	Median follow-up (yr)	Independent variables in the model	Effectiveness of total thyroidectomy for survival
Kenichi Matsuzu, 2013	1088	PTC	17.6	Age, tumor size, extrathyroidal invasion, clinical lymph node metastasis	NA
Iain J. Nixon, 2012	1810	PTC, FTC, HÜrthle cell carcinoma	8.25	Age, male sex,	thyroidectomy vs Lobectomy: HR=0.996 $P = 0.989$
Philip I Haigh, 2005	5432	PTC, FVPTC	7.4	Age, male sex, tumor size, lymph node metastases, lack of radioactive iodine	thyroidectomy vs Lobectomy: Low risk group: HR = 1.73 $P < 0.001$ High risk group: HR = 1.46 $P = 0.14$
Mohamed Abdelgadir Adam, 2014	61775	PTC, FVPTC	6.83	Age, male sex, black race, lower income, tumor size, presence of nodal or distant metastases	thyroidectomy vs Lobectomy: HR = 0.96 $P = 0.54$
Abie H. Mendelsohn, 2010	22724	PTC, FVPTC	9.1	Tumor size, extension, lymph node metastases, age, male sex	Lobectomy vs thyroidectomy: HR = 0.91 $P = 0.41$
Ian D. Hay, 1998		PTC	18	NA	NA
Karl Y. Bilimoria, 2007	52173	PTC	NA	Tumor size	Lobectomy vs thyroidectomy: HR = 1.21 $P = 0.027$
Ernest L. Mazzaferri, 1994	1335	PTC, FTC	15.7	Age, lymph node metastases, tumor size, lack of radioactive iodine	Surgery more than lobectomy vs lobectomy: HR = 0.4 $P > 0.05$

PTC: papillary thyroid carcinoma; FVPTC: follicular variant of papillary thyroid carcinoma; FTC: follicular thyroid carcinoma; NA: not available.
